# Nivolumab-Induced Crescentic Immunoglobulin A Nephropathy With Henoch-Schonlein Purpura Features in a Patient Diagnosed With Hepatocellular Carcinoma

**DOI:** 10.7759/cureus.19110

**Published:** 2021-10-29

**Authors:** Uyioghosa Asemota, Amit Gulati, Kamlesh Kumar, Kalyana Janga

**Affiliations:** 1 Internal Medicine, Maimonides Medical Center, Brooklyn, USA; 2 Nephrology, Maimonides Medical Center, Brooklyn, USA; 3 Nephrology, Maimonides Medical Center, New York, USA

**Keywords:** nivolumab, hepatocellular carcinoma (hcc), ig-a nephropathy, henoch schönlein purpura, crescentic

## Abstract

A 60-year-old Polish male with a history of alcoholism, liver cirrhosis, and hepatocellular carcinoma presented via a referral from his primary medical doctor to the emergency room with respiratory distress, acute kidney injury (AKI), and a purpuric rash on both lower extremities. He had received a total of 16 doses of Nivolumab for hepatocellular carcinoma. He had a baseline serum creatinine of 1.5 and Nivolumab was skipped a month prior to presentation because of a rise in creatinine and the onset of the rash.

Labs showed a blood urea nitrogen (BUN) level of 52 mg/dl and creatinine of 3.2 mg/dl. Urinalysis revealed 300 mg proteinuria and 25-50 red blood cells on a high-power field. He was subsequently placed on steroids for vasculitis manifesting as glomerulonephritis and dermatitis. Biopsy specimens of the kidney and skin were taken and showed focally crescentic diffuse proliferative glomerulonephritis with low-grade A IgA deposits and acute tubular necrosis. The skin biopsy revealed leukocytoclastic vasculitis. We hereby describe a case of focally crescentic diffuse proliferative glomerulonephritis with low-grade A IgA deposits and acute tubular necrosis in an individual with Nivolumab-treated hepatocellular carcinoma.

## Introduction

Nivolumab is an immunoglobulin G4 programmed death-1 (PD-1) immune checkpoint inhibitor monoclonal antibody. PD-1 is an inhibitory transmembrane protein receptor expressed on activated B-cells, T-cells, and NK cells. When PD-1 is bound to programmed death-ligand-1 (PD-L1) on cancer cells, the T-cells become inactivated and the immune response becomes suppressed [1].

PD-1 can be bound to PD-LI or PD-L2, which are expressed by tumor cells and infiltrating immune cells. Inhibiting the interaction between receptor, PD-1, and its ligands, PD-L1 or PD-L2, can enhance the cell-mediated immune response by increasing T-cell activation to promote anti-tumor responses and cancer cell apoptosis and delay the growth of infiltrating immune cells [2].

Immunotherapy (IT) is a rapidly expanding field in oncologic treatments. The use of IT checkpoint inhibition has been promising in treating many cancers, but not without their own unique category of side-effects known as immune-related adverse events (irAE).

There are many cases clearly reported in the literature of renal side effects due to monotherapy with anti-PD-1 drugs.

The recommended dose of Nivolumab is 3.0 mg/kg administered intravenously over 60 minutes every two weeks. Monoclonal antibodies as a class are frequently reported to cause skin toxicity with rashes and pruritus as common findings. Nivolumab use as a single agent has been observed to cause immune-mediated nephritis and renal dysfunction. In terms of kidney function, the clinical course is generally asymptomatic with a gradual increase in creatinine.

## Case presentation

**Figure 1:**
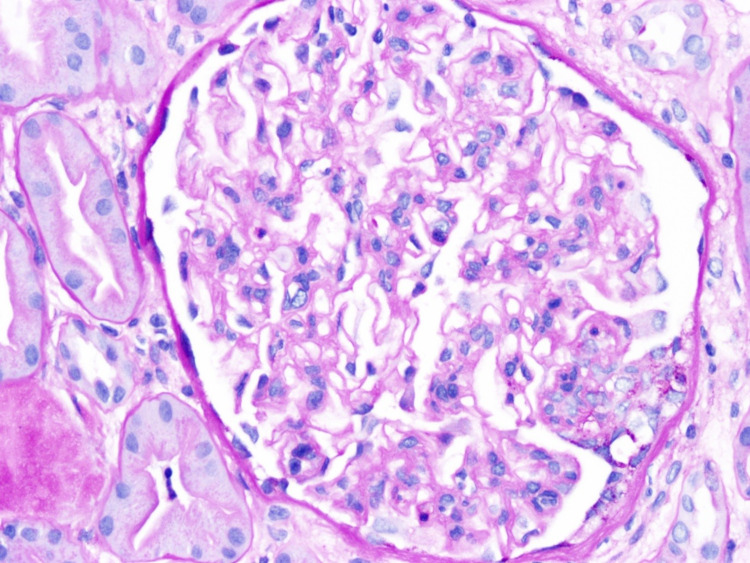
The light microscopy shows diffuse but variable glomerular mesangial matrix expansion along with diffuse moderate mesangial cell proliferation and focal endocapillary cell proliferation. The tubules show a focal dilatation along with epithelial cell injury, necrosis, or flattening. The interstitium demonstrates moderate interstitial fibrosis with no significant interstitial inflammation.

**Figure 2:**
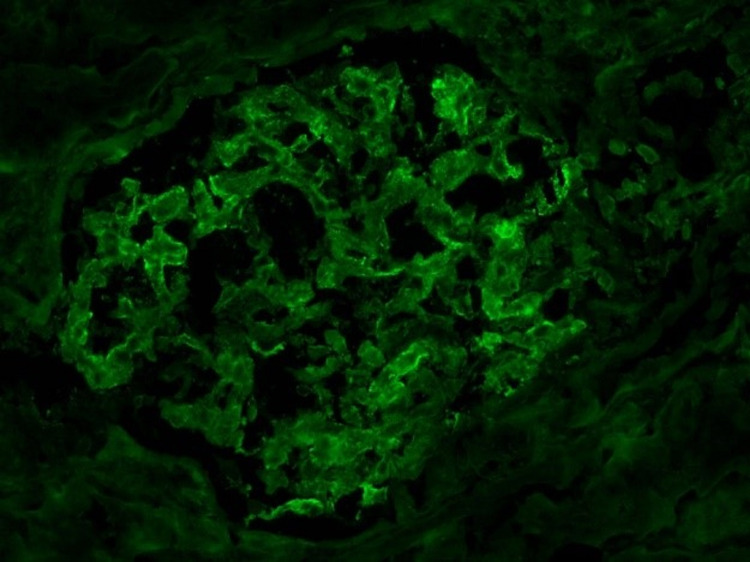
The immunofluorescence analysis demonstrates trace-to-mild diffuse granular glomerular mesangial IgA deposition along with trace IgM and C3 staining. No linear C3 staining of the tubular membranes is apparent. The lambda and kappa light chain staining are heterogeneous (i.e., non-monoclonal) in the IgA staining pattern and involve tubular casts. No significant staining is apparent for IgG, C1q, or fibrinogen.

**Figure 3:**
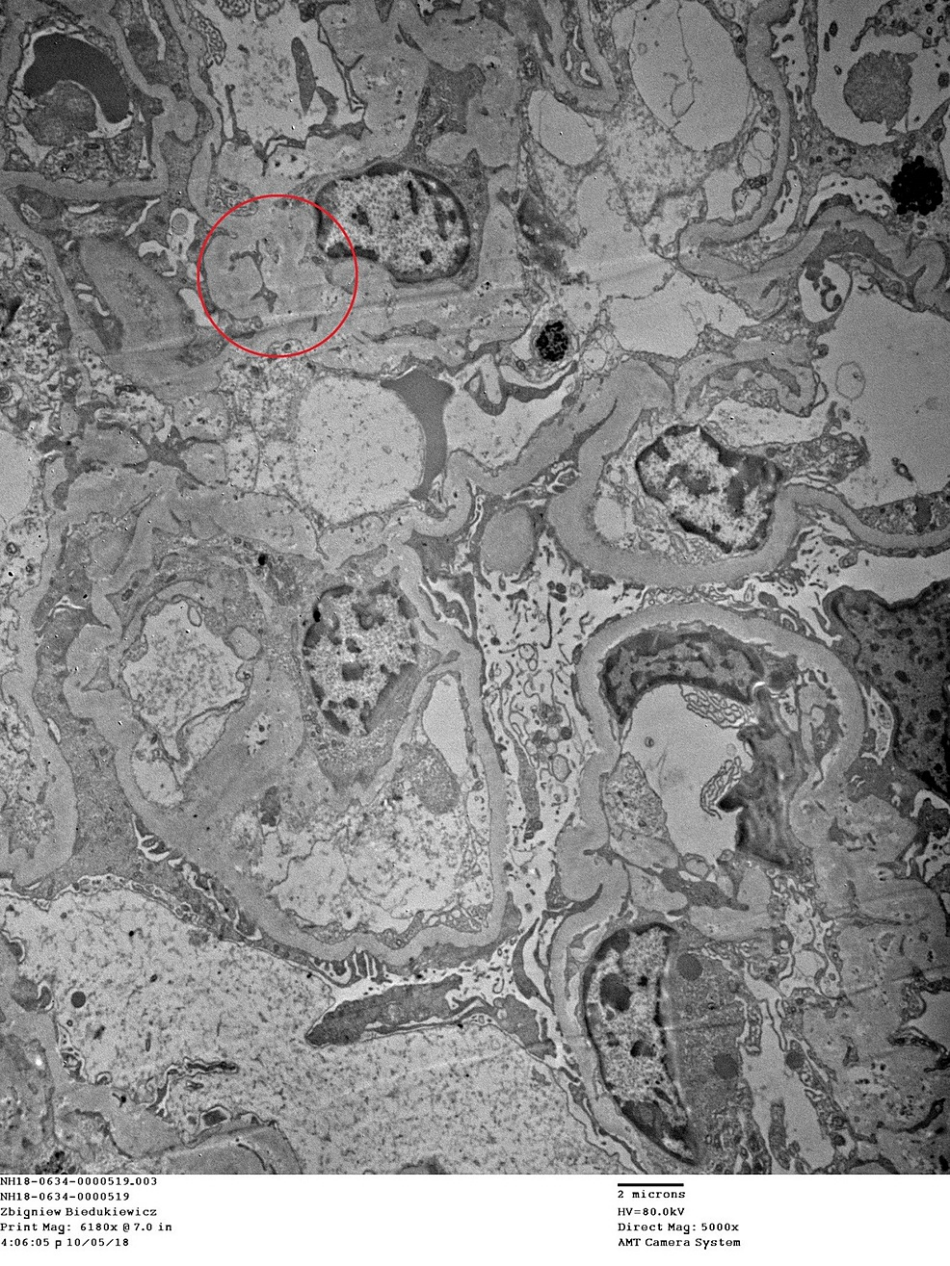
Electron microscopy demonstrates a viable glomerulus with variable mesangial matrix expansion and glomerular basement membrane thickening but no lamination or defects. There are small mesangial deposits apparent. A fibro-cellular glomerular crescent is apparent. Glomerular foot process effacement is segmentally present. The tubules display epithelial cell changes consistent with acute tubular injury.

A 60-year-old Polish male with a history of alcoholism, liver cirrhosis, and hepatocellular carcinoma presented with respiratory distress, purpuric rash on both lower extremities, and acute kidney injury (AKI) via referral from his primary medical doctor. He was on Nivolumab for hepatocellular cancer and had received a total of 16 doses. Nivolumab was, however, skipped a month prior to presentation on account of rising blood urea nitrogen (BUN) and creatinine as well as the onset of the purpuric rash. On physical examination, he was tachypneic but able to sustain a conversation, he had papular, purpuric rash prominent on both lower extremities. Labs showed elevated BUN/creatinine at 52/3.6, baseline creatinine of 1.4, hyperkalemia of 5.1, and metabolic acidosis with bicarbonate of 11, and blood gas PH of 7.12. Urinalysis was positive for proteinuria and microscopic hematuria. Labs were negative for c-ANCA, p-ANCA, atypical ANCA, and complement levels were within normal limits. He was admitted for AKI superimposed on chronic kidney disease based on elevated BUN/creatinine and worsening renal function. Serologic tests were negative including ANCA panel and complement levels were within normal limits. A skin biopsy was done which showed leukocytoclastic vasculitis as well as a kidney biopsy which showed focally crescentic IgA nephropathy (Figures 1-3). He was started on methylprednisolone 1 g daily for three days and subsequently maintained at 1 mg/kg of prednisone. The purpuric rash resolved in response to steroid treatment, but his renal function continued to get worse requiring intermittent hemodialysis. His hospital course was complicated by septic shock, he was on the maximum dose of norepinephrine on the day of his death, 18 days after admission.

## Discussion

This patient presented with symptoms suggestive of AKI and a purpuric rash, raising the suspicion of a vasculitic cause of AKI. He had elevated BUN/creatinine, proteinuria, and hemoglobinuria. Proteinuria is the hallmark of glomerulonephritis and an important predictor of outcome. The etiology could be infectious, immune, drug-related, or idiopathic. The clinical suspicion for this patient was drug-related immune glomerulonephritis. He was started on steroids even before a histological confirmation of the diagnosis of diffuse proliferative glomerulonephritis with IgA deposits.

Glomerulonephritis refers to glomerular diseases with an underlying immune or inflammatory pathogenesis. Clinical manifestations of the glomerular disease are non-specific and include edema, frothy urine, and occasionally progressive decline in renal function. Physical examination findings may include features of fluid retention like pitting pedal edema and pulmonary edema and a hypercoagulable state, especially, in nephrotic syndrome. Proteinuria, hematuria, and cellular casts are common laboratory findings of glomerulonephritis. Biopsy and histology are also essential in the diagnosis and classification of glomerular diseases.

Nivolumab antibody binds to PD-1 and promotes immunity by preventing cancer cell PD-L1 from binding to PD-1 and triggering inactivation of the cell-mediated immunity. Nivolumab is found to be effective for treating non-small cell lung cancer (NSCLC), melanoma, and renal cell carcinoma (RCC). Nivolumab blocks T-cell inhibition and stimulates the immunologic response towards cancer cells, but at the expense of impairing the self-tolerance of the immune system [3].

PD-1 when expressed on activated CD4+ and CD8+ T-cells serves to down-regulate the immune response and promote self-tolerance by suppressing T-cell inflammatory activity. PD-1 is a physiologic immune checkpoint that guards against autoimmunity by promoting apoptosis of antigen-specific T-cells that function to suppress an overactive immune response. Many cancers and viral infections exploit this pathway to achieve evasion of the immune system [4]. One virus that has been shown to exploit this mechanism is the polyomavirus, JC virus, which is responsible for progressive multifocal leukoencephalopathy. Blockage of this pathway with a PD-1 immune checkpoint inhibitor monoclonal activity led to stabilization or clinical improvement in five of eight patients treated with Pembrolizumab [5].

Immune modulators have significantly improved the treatment and prognosis of patients with metastatic cancer. According to a meta-analysis of studies on immune-related adverse events associated with immune checkpoint inhibitors, the overall incidence of irAEs was 26.82% and the incidence of severe grade irAEs was 6.10%. The most common irAEs affect the skin with pruritus and rash is very common. Endocrine, gastrointestinal, liver, and pulmonary disease have all been reported as manifestations of irAEs with the least incidence of organ involvement in the kidneys [6].

However, as the use of Nivolumab in the treatment of metastatic cancer becomes more common, there are increasing reports of renal disease complicating treatment. We report a case of IgA nephropathy developing after the institution of Nivolumab in our patient as has been reported by Tanabe et al. and Kishi et al. [7,8]. Many studies have shown Nivolumab to induce other forms of glomerular injuries [9,10]. It has also been implicated in tubulointerstitial nephritis. In one study, acute tubulointerstitial nephritis was found to be the predominant cause of acute kidney injury in patients treated with Nivolumab. Twelve out of 13 patients who developed AKI in that study had acute tubulointerstitial nephritis (ATIN) [11]. The mechanism is unclear but it is postulated that Nivolumab induces ATIN by altering the immune tolerance intrinsic kidney antigens. Another suggested mechanism of acute interstitial nephritis (AIN) induced by Nivolumab is the reactivation of exhausted drug-specific T-cells primed by prior exposure to nephrotoxic drugs like proton-pump inhibitors (PPIs) and nonsteroidal anti-inflammatory drugs (NSAIDs) [12]. Ashour et al. postulate that reduced self-tolerance, formation of auto-antibodies, deposition of immune complexes, and inflammatory response have a role to play in the development of glomerular injury [13].

Biomarkers have been identified in order to predict the clinical efficacy of immune checkpoint inhibitors and minimize potential side effects. Some of these biomarkers include increased peripheral blood absolute lymphocyte count, increased absolute eosinophil count, increased expression of PD-L1, and clonal mutation of neoantigens.

In clinical practice, irAE have been managed by treatment cessation and systemic corticosteroids as the first line, and use of tumor necrosis factor inhibitors and cytotoxic immunosuppressants as the second line, although the literature is conflicting in whether this second line of management is necessary [14]. Our patient had AKI secondary to Nivolumab-induced glomerulonephritis as well as acute tubular necrosis. The clinical judgment was made at first contact and he was appropriately placed on steroids even before histological confirmation of the clinical diagnosis.

## Conclusions

This case report describes the development of acute glomerulonephritis and acute tubular necrosis in a patient treated with Nivolumab for hepatocellular cancer. There is an increasing awareness of the nephrotoxic potential of Nivolumab and immune checkpoint inhibitors which used to be considered safe for the kidneys. The possible causes of renal failure in this group of patients are multiple, including sepsis, nephrotoxicity, and dehydration, therefore, the threshold for suspicion of an irAE as a cause of renal dysfunction should be low in any patients on immunotherapy.

## References

[1] Darvin P, Toor SM, Sasidharan Nair V, Elkord E (2018). Immune checkpoint inhibitors: recent progress and potential biomarkers. Exp Mol Med.

[2] Guo L, Zhang H, Chen B (2017). Nivolumab as programmed death-1 (PD-1) inhibitor for targeted immunotherapy in tumor. J Cancer.

[3] Spain L, Diem S, Larkin J (2016). Management of toxicities of immune checkpoint inhibitors. Cancer Treat Rev.

[4] Johnson DB, Peng C, Sosman JA (2015). Nivolumab in melanoma: latest evidence and clinical potential. Ther Adv Med Oncol.

[5] Cortese I, Muranski P, Enose-Akahata Y (2019). Pembrolizumab treatment for progressive multifocal leukoencephalopathy. N Engl J Med.

[6] Wang PF, Chen Y, Song SY (2017). Immune-related adverse events associated with anti-PD-1/PD-L1 treatment for malignancies: a meta-analysis. Front Pharmacol.

[7] Tanabe K, Kanzaki H, Wada T, Nakashima Y, Sugiyama H, Okada H, Wada J (2020). Nivolumab-induced IgA nephropathy in a patient with advanced gastric cancer: a case report. Medicine (Baltimore).

[8] Kishi S, Minato M, Saijo A (2018). IgA nephropathy after nivolumab therapy for postoperative recurrence of lung squamous cell carcinoma. Intern Med.

[9] Daanen RA, Maas RJ, Koornstra RH, Steenbergen EJ, van Herpen CM, Willemsen AE (2017). Nivolumab-associated nephrotic syndrome in a patient with renal cell carcinoma: a case report. J Immunother.

[10] Takahashi N, Tsuji K, Tamiya H, Shinohara T, Kuroda N, Takeuchi E (2018). Goodpasture's disease in a patient with advanced lung cancer treated with nivolumab: an autopsy case report. Lung Cancer.

[11] Cortazar FB, Marrone KA, Troxell ML (2016). Clinicopathological features of acute kidney injury associated with immune checkpoint inhibitors. Kidney Int.

[12] Wanchoo R, Karam S, Uppal NN (2017). Adverse renal effects of immune checkpoint inhibitors: a narrative review. Am J Nephrol.

[13] Ashour T, Nakhoul G, Patil P, Funchain P, Herlitz L (2019). Immune check point inhibitor-associated glomerulonephritis. Kidney Int Rep.

[14] Jung K, Zeng X, Bilusic M (2016). Nivolumab-associated acute glomerulonephritis: a case report and literature review. BMC Nephrol.

